# ESBL-Producing *E. coli* in Captive Black Bears: Molecular Characteristics and Risk of Dissemination

**DOI:** 10.3390/vetsci12111085

**Published:** 2025-11-14

**Authors:** Xin Lei, Mengjie Che, Yuxin Zhou, Shulei Pan, Xue Yang, Siyu Liu, Iram Laghari, Mingyue Wu, Ruilin Han, Xiaoqi Li, Lei Zhou, Guangneng Peng, Haifeng Liu, Ziyao Zhou, Kun Zhang, Zhijun Zhong

**Affiliations:** 1 Key Laboratory of Animal Disease and Human Health of Sichuan, College of Veterinary Medicine, Sichuan Agricultural University, Chengdu 611130, China; 15680780217@163.com (X.L.); 15709481145@163.com (M.C.); 13388313209@163.com (Y.Z.); sicaupan@163.com (S.P.); yangx00112@163.com (X.Y.); llsy6412024@163.com (S.L.); 18583228729@163.com (I.L.); 15282103839@163.com (M.W.); han2ruilin@163.com (R.H.); lixiaoqi1982@163.com (X.L.); pgn.sicau@163.com (G.P.); 410140017@163.com (H.L.); zzhou@sicau.edu.cn (Z.Z.); 15281297124@163.com (K.Z.); 2 Sichuan Institute of Musk Deer Breeding, Dujiangyan 611845, China; zhoulei1001@126.com

**Keywords:** *Escherichia coli*, extended-spectrum β-lactamase, β-lactam antibiotics, horizontal gene transfer, black bear

## Abstract

The emergence and global dissemination of extended-spectrum β-lactamase (ESBL)-producing *Escherichia coli* (ESBL-*E. coli*) represent a major public health concern. However, the characterization and capacity for horizontal gene transfer (HGT) of ESBL-*E. coli* in captive black bears remain substantially understudied. In the present study, 19 ESBL-*E. coli* strains were successfully identified (13.38%, 19/142). A total of 11 sequence types (STs) were identified from 19 ESBL-*E. coli* strains using MLST. This included eight known types (ST10, ST2690, ST208, ST695, ST4160, ST540, ST3865 and ST2792) and three new STs. Antimicrobial susceptibility testing demonstrated that all 19 ESBL-*E. coli* exhibited high resistance to KZ (100.00%), CRO (78.95%), and CTX (73.68%). Polymerase chain reaction (PCR) screening for 14 β-lactam antibiotic resistance genes (ARGs) and their variants revealed that *bla*_CTX-M_ was the most prevalent, followed by *bla*_SHV_, *bla*_TEM_, and *bla*_DHA_. Furthermore, eight β-lactamase variants were detected, including five *bla*_CTX-M_ variants (*bla*_CTX-M-15_, *bla*_CTX-M-3_, *bla*_CTX-M-14_, *bla*_CTX-M-55_, and *bla*_CTX-M-27_) and one variant each of *bla*_SHV-1_, *bla*_TEM-1_, and *bla*_DHA-14_. Conjugation assays revealed that eight ESBL-*E. coli* strains were capable of conjugative transfer. Five plasmid types (IncFII, IncW, IncFrepB, IncY, and IncHI1) and three mobile genetic elements (MGEs) (*IS26*, *ISEcp1*, and *trbC*) were identified as co-transferred with *bla*_CTX-M_. ESBL-*E. coli* poses a potential threat to captive black bears and may lead to further transmission. Consequently, the implementation of continuous surveillance and targeted interventions is imperative to prevent the transmission of ESBL-*E. coli*.

## Simple Summary

The emergence of resistance to β-lactam antibiotics and the characteristics of extended-spectrum β-lactamase (ESBL)-producing *Escherichia coli* (ESBL-*E. coli*), represent a significant concern in both human and veterinary medicine. Wildlife is recognized as a reservoir of ESBL-*E. coli*, but the role that wildlife plays in the dissemination of antimicrobial resistance genes (ARGs) is still not fully understood. In the present study, we report the first identification of ESBL-*E. coli* in captive black bears and reveal a high prevalence of β-lactam resistance. Conjugative transfer assays have demonstrated that these bacteria exhibit high transfer efficiency, and have further demonstrated that multiple ARGs, mobile genetic elements (MGEs), and plasmids were capable of horizontal transmission. These findings indicate that wildlife serves as a reservoir for antibiotic-resistant bacteria with dissemination potential, underscoring the critical importance of antimicrobial resistance (AMR) monitoring and the implementation of a One Health approach.

## 1. Introduction

*Escherichia coli* (*E. coli*) is a Gram-negative bacterium that belongs to the *Enterobacteriaceae* family. *E. coli* is a commensal bacterium that is commonly found in the intestines of humans and animals [1]. Among the antibiotics available on the market, β-lactam antibiotics are the most dominant antimicrobial agents globally, accounting for approximately 50% of all prescribed antimicrobial agents [2]. The misuse of clinical antimicrobials exerts selective pressure on bacteria, causing them to evolve or acquire antimicrobial resistance genes (ARGs), thereby developing antimicrobial resistance (AMR). AMR poses a significant challenge in the treatment of bacterial infections in both humans and animals [3]. Furthermore, the presence of extended-spectrum β-lactamase (ESBL)-producing *E. coli* (ESBL-*E. coli*) represents a significant global public health threat [4]. ESBLs represent a primary cause of the failure of β-lactam antibiotic treatment [5]. Consequently, the study of resistance to β-lactam antibiotics and the characteristics of ESBL-*E. coli* has attracted scholarly interest, particularly in the context of veterinary clinical practice [6,7].

Over the past decade, ESBLs, AmpC β-lactamases and carbapenemases have been documented in *Enterobacteriaceae* originating from both humans and animals [8]. These enzymes have been demonstrated to confer bacterial AMR to a wide range of β-lactam antibiotics, including penicillins, cephalosporins and carbapenems [9]. In addition, among these enzymes, ESBL-*E. coli* is the most frequently isolated from wild animals and can disseminate rapidly via horizontal gene transfer (HGT) [10,11]. The horizontal transfer of ESBL-resistant genes in ESBL-*E. coli* is mainly driven by mobile genetic elements (MGEs), including plasmids, transposons, and insertion sequences (ISs) [12,13]. Animals are recognized as potential reservoirs for transmitting AMR bacteria to humans; consequently, bacteria harboring β-lactam AMR pose a serious public health threat [8].

The Asian black bear (*Ursus thibetanus*) serves as a keystone component of ecosystems, playing critical roles in maintaining ecological balance and stability [14]. In China, there are five subspecies of the Asiatic black bear (*U. t. thibetanus*, *U. t. laniger*, *U. t. mupinensis*, *U. t. formosanus*, and *U. t. ussuricus*), and the Sichuan subspecies (*U. t. mupinensis*) is the most widely distributed black bear in China [15]. In the field of wildlife, the first documented cases of ESBL-*E. coli* were observed in deer, owls, bird of prey and foxes in Portugal in 2006 [16]. Subsequent to the initial detection, the presence of ESBL-*E. coli* has been identified in a variety of wildlife species, including cave bats, feral swine, Canis latrans, Orangutan, and giant pandas [17,18,19,20]. Notably, the ESBL-*E. coli* is one of the most important pathogens at the One Health interface [21]. To date, only two studies have analyzed ESBL-*E. coli* in bears. A study successfully isolated 17 ESBL-*E. coli* from Indian sloth bears [22]. Another study isolated only one ESBL-*E. coli* from Eurasian brown bears in Spain [23]. ESBL-*E. coli* has not been identified from captive black bears in China, and its HGT potential remains uncharacterized, which underscores the necessity of this investigation.

Our previous study demonstrated a high prevalence of AMR among 142 *E. coli* isolates from captive black bears (*U. t. mupinensis*), 65.49% (93/142) of isolates exhibiting resistance to β-lactam antibiotics [24]. In the present study, we performed β-lactam resistance phenotyping, ARG profiling, and an assessment of the HGT potential on ESBL-*E. coli* isolates from 142 *E. coli* isolates to better understand the characterization of ESBL-*E. coli* from captive black bears.

## 2. Materials and Methods

### 2.1. Bacterial Strains

The present study utilized 142 *E. coli* strains isolated from captive black bear fecal samples, with all strains preserved in the laboratory prior to analysis [24]. The 142 fecal samples were collected from a black bear breeding farm in Dujiangyan City, China (103.59° E, 31.02° N), with one sample obtained per individual.

### 2.2. Screening of ESBL-E. coli Isolates

ESBL-*E. coli* isolates from captive black bears were screened using the double-disk synergy test, following the CLSI 2023 guidelines. The antimicrobial susceptibility disks employed were ceftazidime (CAZ), ceftazidime-clavulanate (CAL), cefotaxime (CTX), and cefotaxime-clavulanate (CTL). The isolates were cultured on Mueller-Hinton agar plates and incubated at 37 °C for 16–18 h. After incubation, the zones of inhibition were measured. An isolate was confirmed as ESBL-producing if the zone diameter for either combination disk (CAL or CTL) showed a ≥5 mm increase compared to its corresponding β-lactam alone disk (CAZ or CTX, respectively).

### 2.3. MLST Typing of ESBL-E. coli Isolates

MLST was performed by amplifying of seven housekeeping genes (*adk*; *fumC*; *gyrB*; *icd*; *mdh*; *purA* and *recA*) according to the standard protocol on PubMLST (https://pubmlst.org/organisms/escherichia-spp (accessed on 10 October 2025)) [25]. The Primer sequences and amplification parameters are detailed in Supplementary Table S1. The positive PCR products were submitted for bidirectional sequencing at Sangon Biotech (China). The bidirectional sequencing reads for each ESBL-*E. coli* isolate were assembled and aligned against the *E. coli* MLST database (https://pubmlst.org/ (accessed on 4 November 2025)) to determine their sequence types (STs). Using the goeBURST algorithm in Phyloviz 2.0, clonal clustering analysis assigned STs of successfully typed ESBL-*E. coli* isolates to distinct clonal complexes (CCs), where each CC comprises closely related STs sharing ≤2 allelic differences.

### 2.4. Antimicrobial Susceptibility Testing for ESBL-E. coli Isolates

All ESBL-*E. coli* isolates underwent standardized disk diffusion susceptibility testing against 15 β-lactam antibiotics representing five classes. A total of seven antibacterial agents were tested in our previous study, including ampicillin (AMP), cephalosporins (KZ, CXM, CTX, FEP), monobactams (ATM) and β-lactamase inhibitor combinations (SAM) [24]. The remaining eight antibacterial agents were tested in this study: cephalosporins (cefoxitin, FOX, 30 μg; ceftriaxone, CRO, 30 μg; ceftazidime, CAZ, 30 μg), carbapenems (imipenem, IMP, 10 μg; meropenem, MEM, 10 μg; Ertapenem, ETP, 10 μg), β-lactamase inhibitor combinations (piperacillin/tazobactam, TZP, 100/10 μg; amoxicillin/clavulanic acid, AMC, 20/10 μg). Susceptibility results were interpreted using CLSI 2023 breakpoint criteria. *E. coli* ATCC25922 was used as the quality control strain. The antibiotics used in this study were based on the information from local farm veterinarians on which antibiotics were used for disease control (CTX, CXM and SAM); AMP, KZ, FEP, FOX, CRO, CAZ, ATM, IMP, MEM, ETP, TZP and AMC have been reported to be found antibiotic resistant in wildlife [26,27,28].

### 2.5. Screening of β-Lactam ARGs and MGEs from ESBL-E. coli Isolates and Outer Membrane Proteins from ESBLs16

Genomic DNA from ESBL-*E. coli* isolates was extracted using a Tiangen Biotech kit (Beijing, China) according to the manufacturer’s protocol. The purity and concentration of the DNA were verified by measuring the A260/A280 absorption ratio. Extracted DNA was stored at −20 °C for subsequent PCR amplification. Based on relevant studies, 14 β-lactam ARGs were selected for detection: the ESBL genes bla_TEM_, bla_SHV_ and bla_CTX-M_; the AmpC genes bla_MOX_, bla_CIT_, bla_DHA_, bla_ACC_, bla_EBC_ and bla_FOX_; and the carbapenemase genes bla_KPC_, bla_NDM-1_, bla_IMP_, bla_VIM_ and bla_SEM_ [29,30,31,32]. As bla_CTX-M_ had been characterized in prior experiments [24], bla_CTX-M_-positive isolates were selected for molecular subtyping to identify genetic subgroups (groups 1, 2, 8, 9, and 25) [29]. We performed PCR amplification of the OmpC and OmpF genes in isolate ESBLs16 to assess potential deficiencies in them [33]. Full primer sequences and amplification parameters are detailed in Supplementary Table S1. The positive PCR products were submitted for unidirectional Sanger sequencing at Sangon Biotech (China). The obtained sequences were aligned against ARG sequences in the GenBank database using the BLASTn algorithm on the NCBI website to assess sequence similarity and identify target fragments (https://blast.ncbi.nlm.nih.gov/Blast.cgi (accessed on 4 November 2025)). The data concerning MGEs for the 19 ESBL-*E. coli* were obtained from a previous study [24].

### 2.6. Data of AMR, ARGs, and MGEs Analyzed and Association Analysis Between AMR and ARGs or MGEs

The data on AMR, ARGs, and MGEs were analyzed by using SPSS software (version 26.0). Associations among AMR phenotypes, ARGs, and MGEs were analyzed using Spearman’s correlation test, with a *p*-value < 0.05 considered statistically significant. The results were visualized using the ggplot2 package in RStudio (version 4.2.2) [34].

### 2.7. Conjugation Assays and PCR-Based Replicon Typing (PBRT)

To investigate the conjugative transfer of β-lactam ARGs and associated MGEs, 19 ESBL-*E. coli* strains were used as donors, with sodium azide-resistant *E. coli* J53 as the recipient. The donor and recipient strains were separately cultured in 4.0 mL of Luria–Bertani (LB) broth at 37 °C for 16 h. Then, 0.2 mL of the donor culture was mixed with 0.8 mL of the recipient culture in 4.0 mL of fresh LB broth. A control contained 0.8 mL recipient culture in 4.2 mL LB broth. Both mixtures were incubated at 37 °C for 16 h. Transconjugants were selected and quantified on LB agar plates supplemented with sodium azide (100 μg/mL) and CTX (4 μg/mL), while recipient cell counts were determined on azide-containing plates (100 μg/mL) without antibiotic supplementation. The conjugation frequency was calculated as the number of transconjugants divided by the number of recipient cells, thus representing the HGT frequency. The ESBL phenotype of transconjugants was confirmed by the double-disk synergy testing, and transferred ARGs and MGEs were detected by PCR [35]. Plasmid replicon types were determined by PCR using 18 primer pairs specific for replicon typing (IncHI1, IncHI2, IncI1, IncX, IncL/M, IncN, IncFIA, IncFIB, IncW, IncY, IncP, IncFIC, IncA/C, IncT, IncFII, IncFrepB, IncK/B, IncK and IncB/O) [36]. The subsequent analysis of the PCR products was conducted in accordance with the methodology delineated in Section 2.5. The primer sequences and corresponding amplification conditions are provided in Supplementary Table S1.

## 3. Results

### 3.1. ESBL Strains Identified and MLST Analysis

**Figure 1:**
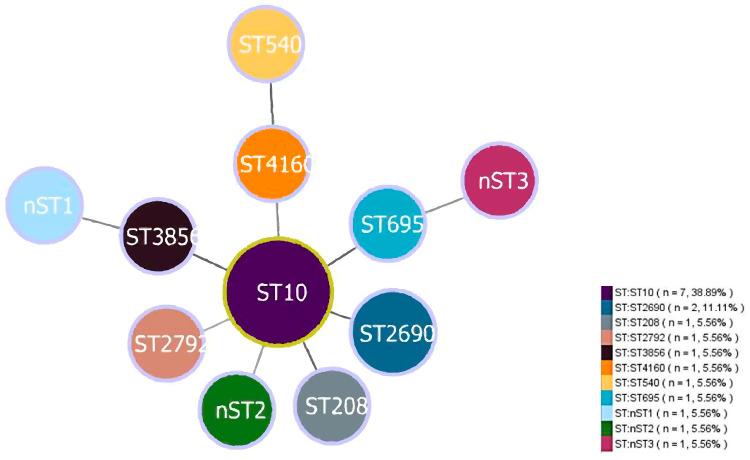
Minimum spanning tree of MLST types in 18 ESBL-*E. coli* strains. The size of circle indicates the proportion of isolates belonging to the ST. The yellow outline of the circle represents the adjacent STs belonging to the same clonal complex.

Of the 142 *E.coli* isolates from captive black bears, 19 (13.38%, 19/142) were successfully confirmed as ESBL-*E. coli*. Among the 19 ESBL-*E. coli* strains examined, 18 strains (with the exception of ESBLs15) successfully amplified all seven housekeeping genes (Table 1). MLST analysis revealed that 18 isolates exhibited eight distinct STs (Figure 1). ST10 (38.89%, 7/18) was the most prevalent ST, followed by ST2690 (11.11%, 2/18). The remaining six STs contained only a single strain each. Furthermore, three new STs (designated nST1 to nST3) were identified. Using the goeBURST algorithm, only one clonal complex (CC10) was identified among the 15 ESBL-*E. coli* isolates that were successfully assigned an ST from the database. This complex comprised eight isolates (53.33%), while the remaining seven isolates were not assigned to any clonal complex.

### 3.2. The Phenotype of Resistance to β-Lactam Antibiotics

**Figure 2:**
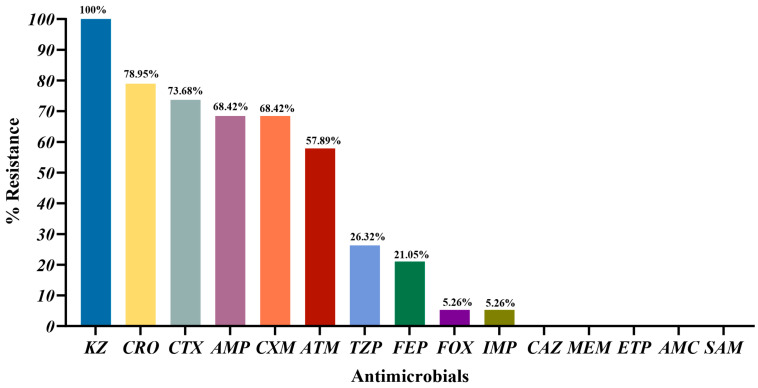
Percentage of antibiotic resistance in 19 ESBL-*E. coli*. KZ-cefazolin; CRO-ceftriaxone; CTX-cefotaxime; AMP-Ampicillin; CXM-cefuroxime; ATM-aztreonam; TZP-piperacillin/tazobactam; FEP-cefepime; FOX-cefoxitin; IMP-imipenem; CAZ-ceftazidime; MEM-meropenem; ETP-ertapenem; AMC-amoxicillin/clavulanic acid; SAM-ampicillin/sulbactam.

As shown in Figure 2, the highest resistance rate was observed for KZ (100%, 19/19), followed by CRO (78.95%, 15/19) and CTX (73.68%, 14/19). The resistance rates for the remaining β-lactam antibiotics ranged from 68.42% (AMP, CXM) to 21.05% (FEP). All 19 ESBL-*E. coli* isolates were susceptible to CAZ, MEM, ETP, AMC, and SAM. The susceptibility rates for FOX and IMP were all 94.74% (18/19).

**Figure 3:**
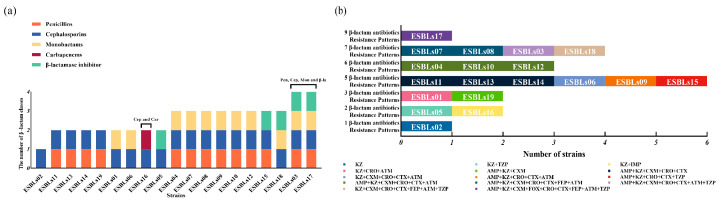
The resistance patterns of ESBL-*E. coli*. (**a**) Color bar chart represents the resistance patterns of ESBL-*E. coli* to five classes of β-lactam antibiotics. Each color represents a distinct class of β-lactam antibiotics. A total of 9 resistance patterns were observed. Pen-Penicillins; Cep-Cephalosporins; Mon-Monobactams; Car-Carbapenems; β-la-β-lactamase inhibitor. (**b**) Color bars demonstrate the distribution of phenotypic resistance patterns to 15 β-lactam antibiotics among ESBL-*E. coli* (*n* = 19). The specific strain designations are illustrated in the color bars a total of 14 resistance patterns were observed by using disk diffusion assay.

As illustrated in Figure 3a, the nine resistance patterns are distributed across five classes of β-lactam antibiotics. All 19 ESBL-*E. coli* isolates exhibited resistance to cephalosporins. It is noteworthy that strains ESBLs03 and ESBLs17 were resistant to all tested β-lactam classes with the exception of carbapenems. Isolate ESBL16 was the only strain exhibiting resistance to carbapenems. As illustrated in Figure 3b, the 14 resistance patterns to 15 β-lactam antibiotics are demonstrated. All 19 ESBL-*E. coli* isolates exhibited resistance to at least one β-lactam antibiotic. In particular, isolate ESBLs17 exhibited resistance to nine distinct β-lactam antibiotics (AMP/KZ/CXM/FOX/CRO/CTX/FEP/ATM/TZP). The most prevalent pattern (31.58%, 6/19) was resistance to five antibiotics (AMP/KZ/CXM/CRO/CTX, KZ/CXM/CRO/CTX/ATM, AMP/KZ/CRO/CTX/ATM, and AMP/KZ/CRO/CTX/TZP).

### 3.3. Prevalence of MGEs and ARGs in 19 ESBL-E. coli Isolates and OMPs in ESBLs16

Table 2 presents the detection rates of β-lactam ARGs among the 19 ESBL-*E. coli* isolates. A total of four out of the 14 target ARGs were detected. The highest detection rate was for *bla*_CTX-M_ (78.95%, 15/19), followed by *bla*_SHV_ (10.53%, 2/19). *bla*_TEM_ and *bla*_DHA_ were only detected in one strain (5.26%, 1/19), respectively. In contrast, genes encoding for AmpC β-lactamases (*bla*_MOX_, *bla*_CIT_, *bla*_ACC_, *bla*_EBC_, *bla*_FOX_) and carbapenemases (*bla*_KPC_, *bla*_NDM-1_, *bla*_IMP_, *bla*_VIM_, *bla*_SEM_) were not detected in the 19 isolates. Subsequent analysis was conducted in order to characterize the subtypes of the β-lactamase genes (*bla*_CTX-M_, *bla*_SHV_, *bla*_TEM_ and *bla*_DHA_). Five variants of *bla*_CTX-M_ were identified. Furthermore, the presence of one variant each of *bla*_SHV-1_, *bla*_TEM-1_, and *bla*_DHA-14_ was detected. Among the five *bla*_CTX-M_ variants, three belonged to the CTX-M-1 group (*bla*_CTX-M-55_, *bla*_CTX-M-15_ and *bla*_CTX-M-3_) and two to the CTX-M-9 group (*bla*_CTX-M-14_ and *bla*_CTX-M-27_). *bla*_CTX-M-15_ was the most prevalent variant (58.82%, 10/17), followed by *bla*_CTX-M-3_ (17.65%, 3/17), *bla*_CTX-M-14_ (11.76%, 2/17), *bla*_CTX-M-27_ (5.88%, 1/17), and *bla*_CTX-M-55_ (5.88%, 1/17). It is noteworthy that both ESBLs14 (co-harboring *bla*_CTX-M-15_ and *bla*_CTX-M-14_) and ESBLs18 (co-harboring *bla*_CTX-M-3_ and *bla*_CTX-M-14_) contained two coexisting *bla*_CTX-M_ variants.

**Figure 4:**
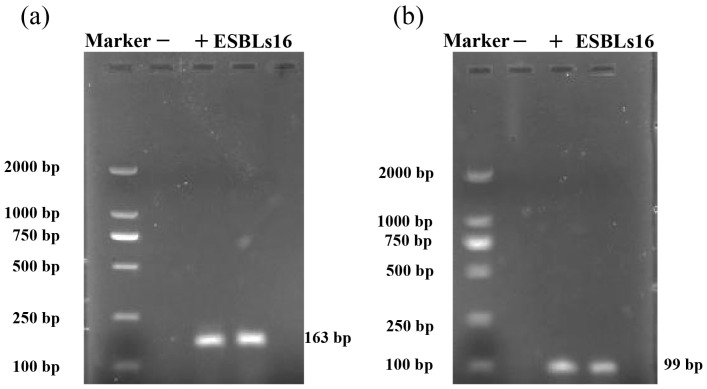
PCR amplification of OmpC and OmpF genes from isolate ESBLs16. (**a**) OmpC of isolate ESBLs16. (**b**) OmpF of isolate ESBLs16. “−” indicates the negative control, and “+” indicates the positive control.

As shown in Figure 4, *OmpC* and *OmpF* were successfully amplified from isolate ESBLs16, indicating that the two genes exist in the ESBLs16 strain.

### 3.4. Associations Between AMR and ARGs or MGEs in 19 ESBL-E. coli Isolates

**Figure 5:**
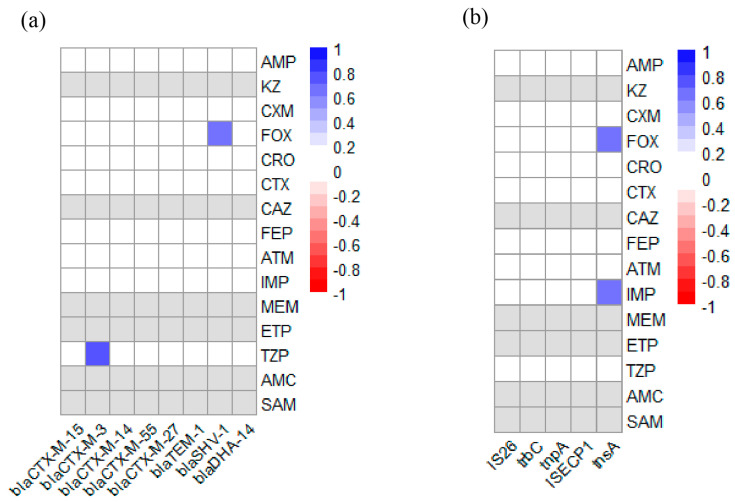
Heatmap of the correlation-coefficient (r) between AMR and ARGs or MGEs in 19 ESBL-*E. coli* strains from black bear. Blue indicates positive association (r > 0, *p* < 0.05) and red indicates negative association (r < 0, *p* < 0.05). Gray indicates that the resistance rate to the antibiotic was either 0% or 100%; therefore, a valid correlation coefficient could not be computed. The color scale on the right of figure indicates the r-valve: (**a**) Heatmap of the correlation coefficient between AMR and ARGs. The color scale and corresponding r-valve indicate the association between corresponding abscissa ARGs and ordinate AMR. (**b**) Heatmap of correlation coefficient between AMR and MGEs. The color scale and corresponding r-valve indicate the association between corresponding MGEs (abscissa) and AMR (ordinate).

We analyzed the associations between AMR phenotypes and ARGs in 19 ESBL-*E. coli* strains. As shown in Figure 5a, two positive association pairs (r > 0, *p* < 0.05) were observed between AMR and ARGs, specifically between the TZP resistance phenotype and the bla_CTX-M-3_ gene, and between the FOX resistance phenotype and the bla_SHV-1_ gene. Furthermore, analysis of the associations between AMR phenotypes and MGEs revealed two additional positive correlations (Figure 5b), involving both the IMP and FOX resistance phenotypes with the *tnsA* gene.

### 3.5. Conjugative Transfer Assays and Replicon Typing

**Figure 6:**
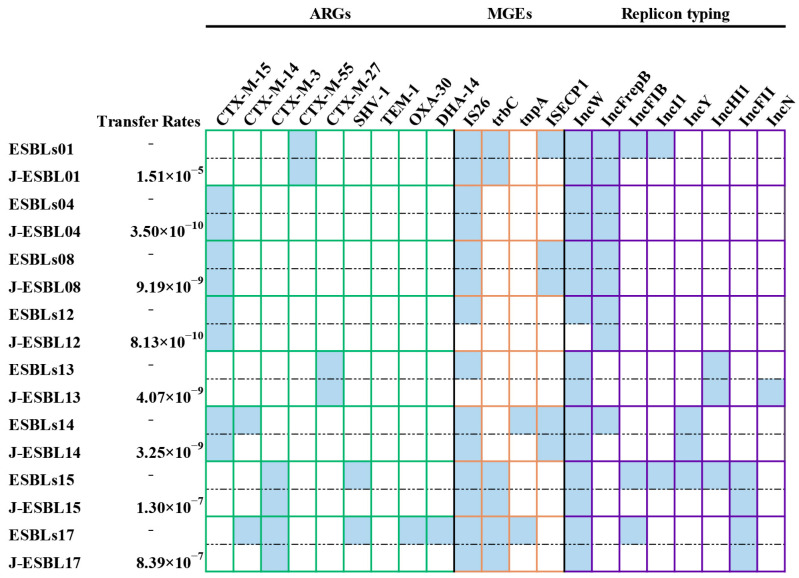
A heatmap comparing ARGs, MGEs, and plasmid replicon types among 8 ESBL-*E. coli* donor strains and their corresponding transconjugants. “J” represents that the strain is a transconjugant. Blue squares represent donor bacteria or transconjugants carrying the gene or plasmid.

We further investigated the conjugative transferability of ARGs, MGEs and plasmids. In the present study, 19 ESBL-*E. coli* strains were examined, of which eight (42.11%, 8/19) were found to be capable of conjugative transfer, with frequencies ranging from 8.13 × 10^−10^ to 1.15 × 10^−5^. All eight transconjugants were confirmed to have the ESBL-producing phenotype. The CTX-M-9 group variants from isolates ESBLs14 and ESBLs17 exhibited no transferability, whereas other *bla*_CTX-M_ variants were successfully transferred to the *E. coli* J53 recipient. Furthermore, *bla*_SHV_, *bla*_TEM_ and *bla*_DHA_ were not detected in any transconjugants. Figure 6 shows the dissemination details of ARGs, MGEs and plasmids for the eight ESBL-*E. coli* donor isolates and transconjugants. Among them, *trbC* was detected in all transconjugants, whereas *tnpA* was not transferred. The transfer of *IS26* was successful in all isolates except for ESBLs12 and ESBLs13. Similarly, *ISEcp1* was transferred successfully in all isolates except for ESBLs1.

PBRT identified eight distinct plasmid types in eight ESBL-*E. coli* isolates, including IncW (100%, 8/8), IncFrepB (62.5%, 5/8), IncFIB (37.5%, 3/8), IncI1 (25%, 2/8), IncY (25%, 2/8), IncHI1 (25%, 2/8), IncFII (25%, 2/8), and IncN (12.5%, 1/8). PBRT analysis of the transconjugants confirmed that five plasmid types (IncFII, IncW, IncFrepB, IncY, and IncHI1) were successfully transferred, while IncFIB, IncI1, and IncN were not observed.

## 4. Discussion

AMR poses one of the most serious global public health threats to both animals and humans in the 21st century [37]. The global emergence of ESBL-*E. coli* represents a significant concern because few antibiotics remain active against such bacteria [38,39]. ESBLs confer resistance to a broad spectrum of β-lactam antibiotics, including penicillins, third-generation cephalosporins, and monobactams, thereby complicating the treatment of infections caused by ESBL-*E. coli* in animals [40]. To date, the presence of ESBL-*E. coli* has been detected in various wildlife species, including gulls, deer, iguana and coatis [41,42,43,44]. However, reports of ESBL-*E. coli* in captive black bears remain limited. Only two studies on Indian sloth bears [22] and Spanish Eurasian brown bears [23] have reported ESBL-*E. coli*. To better understand the characteristics of ESBL-*E. coli* in captive black bears, 142 fecal isolates preserved in our laboratory were analyzed. Our results showed that 19 ESBL-*E. coli* strains were successfully identified from 142 *E. coli* strains (13.38%). The prevalence of ESBL-*E. coli* from captive black bears was similar to the prevalence from Italian wild boars (15.96%) and wild birds (13.2%) [45,46], but lower than that from captive non-human primates in China (32%) [47]. In contrast, the prevalence of ESBL-*E. coli* from captive black bears was higher than that reported from wildlife in Portugal (3%) [48].

MLST is a common method employed in the field to differentiate genetic relatedness based on the analysis of allelic profile similarities [49]. In the present study, ST10 was the most prevalent ST, which was consistent with the detection profiles of ESBL-*E. coli* STs from sheep and primates [50,51]. ST10 is a high-risk clone with a broad host range [52], including livestock (pigs, cattle, sheep and poultry), wildlife (Siberian tigers, North China leopards, and silver gulls), and companion animals (cats and dogs) [53,54,55,56,57,58]. ST10 is also believed to be associated with human-associated infections and has the potential to cause zoonotic transmission [50,59]. The identification of ST10 from captive black bears suggests that continuous monitoring is required to better understand its transmission. In our study, ST2690 was detected as the second prevalent ST (11.11%, 2/18). ST2690 was first identified in ESBL-*E. coli* from domestic ducks [60]. A recent study has also detected ST2690 in ESBL-*E. coli* isolates from captive giant panda breeding environments and their animal keepers [30]. This study demonstrated that ST2690 exhibits conjugation capability, thus indicating its potential to spread to giant pandas [30]. The above results imply that ST2690 was co-circulating among the environment, animals, and humans, indicating a significant risk to public health. Furthermore, six additional known STs (ST540, ST4160, ST2792, ST208, ST695, and ST3856) were also detected in ESBL-*E. coli* from captive black bears. ST540 has been reported in ESBL-*E. coli* from both wild game meat and wild birds in Switzerland [61,62]. Similarly, ST4160 was identified in equine ESBL-*E. coli* in the Netherlands [63], while ST2792 was found in ESBL-*E. coli* from retail chicken meat in Japan [64]. In China, ST208 has been detected in *E. coli* from retail duck meat [65], and ST3856 was the predominant ST in duck-derived *E. coli* [66]. Furthermore, ST695 has been identified in *E. coli* from wild pigeons and penguins in Australia [67]. In summary, ST540, ST4160, ST2792, ST208, ST695, and ST3856 were first detected in ESBL-*E. coli* from captive black bears, highlighting the necessity for sustained wildlife surveillance.

ESBL-*E. coli* is characterized by its resistance to β-lactam antibiotics [68]. Our results showed that the highest resistance rate was observed for KZ, CRO and CTX. KZ (a first-generation cephalosporin) has been used clinically for a period exceeding four decades [69]. All 19 ESBL-*E. coli* isolates were resistant to KZ, which is consistent with the observations reported by Li et al. in ESBL-*E. coli* from broilers [70]. CRO and CTX are widely used third-generation cephalosporins in clinical practice [71]. In our study, the resistance rates to CRO and CTX were 78.95% and 73.68%, respectively. One study reported that ESBL-*E. coli* isolated from pigs exhibited high resistance rates to both CRO and CTX [72], which were similar to the resistance rates observed in our study. The high resistance observed to CRO and CTX may be attributable to the treatment of sick captive black bears with these antibiotics (CRO and CTX) during captive management (information provided by the local farm veterinarians for captive black bears). In our study, ESBL-*E. coli* isolates exhibited strong AMR and a variety of resistance patterns, implying that drug rotation strategies should be considered in the clinical use of antibiotics. These measures aim to reduce the emergence and transmission of AMR bacteria across humans, animals, and environment [73]. Moreover, two ESBL-*E. coli* isolates (ESBLs03 and ESBLs17) exhibited concomitant resistance to four classes of β-lactam antibiotics, yet remained susceptible to carbapenems. The activity of β-lactamases is suppressed by β-lactamase inhibitors, and the combination of these inhibitors with β-lactam antibiotics helps to preserve and extend the latter’s therapeutic efficacy [74,75]. The concomitant resistance to four classes exhibited by ESBLs03 and ESBLs17 implies that treatment options would be severely limited. It is noteworthy that one ESBL-*E. coli* (ESBLs16) was resistant to imipenem despite not carrying any carbapenemase genes. Research has demonstrated that imipenem resistance can emerge in non-carbapenemase-producing *E. coli* strains exhibiting a deficiency in major outer membrane proteins (OMPs) *OmpF* and *OmpC* [76]. Consequently, we employed the PCR to amplify the *OmpF* and *OmpC* genes in strain ESBLs16. The results of the study demonstrated the presence of both *OmpF* and *OmpC* in isolate ESBLs16. The observed imipenem resistance in strains without *OmpF* or *OmpC* deficiencies suggests that further investigation is required into potential resistance mechanisms [77]. In summary, ESBL-*E. coli* from captive black bears demonstrates resistance to various β-lactam antibiotics. This study provides valuable insights that can inform the clinical treatment of infectious diseases in captive black bears.

To date, over 350 natural ESBL variants have been identified and classified into nine structural and evolutionary families (TEM, SHV, CTX-M, PER, VEB, GES, BES, TLA, and OXA) [78]. Among these ESBL variants, CTX-M, TEM, and SHV are frequently detected in wildlife species, including wild boars, chimpanzees, mouflons, and ostriches [45,79]. In our study, CTX-M was the predominant type (78.95%), although its detection rate was lower than that reported in Indian sloth bears (100%) [22] and captive giant pandas (88.9%) [30], yet higher than that in captive wild birds (65.4%) [80]. The detection rates of SHV and TEM in our study were 10.53% and 5.26%, respectively. These rates were found to be significantly lower than those reported in wild bird populations [80,81]. Furthermore, among *bla*_CTX-M_ variants, *bla*_CTX-M-15_ was the most prevalent variant (58.82%, 10/17) in our study. The presence of *bla*_CTX-M-15_, which has been primarily identified in human and veterinary clinical samples, has recently been reported in wildlife [82]. In China, *bla*_CTX-M-15_ has also been detected in wildlife, including giant pandas (8.82%), swans (21.43%), and primates (55.36%) [30,47,83]. The results obtained demonstrate that the *bla*_CTX-M-15_ variant is prevalent among ESBL-*E. coli* in wildlife in China. In the present study, the *bla*_CTX-M-14_ (11.76%, 2/17) variant was also detected in a sample of captive black bears. The *bla*_CTX-M-14_ variant is the most common among ESBL-*E. coli* strains from humans [84]. The presence of *bla*_CTX-M-14_ variants in captive black bears in this study suggests that transmission of these variants via animal keepers is a possibility [85].

The uncontrolled dissemination of plasmids carrying ARGs poses a significant public health threat due to rapid HGT [86]. In the present study, four out of the eight ARGs were successfully transferred (including *bla*_CTX-M-15_, *bla*_CTX-M-3_, *bla*_CTX-M-55_, and *bla*_CTX-M-27_). The remaining ESBL genes were not transferred, a phenomenon that can be attributed to their predominant chromosomal location, a feature that favors vertical transmission [87]. It is noteworthy that among isolates ESBLs14 and ESBLs18, the *bla*_CTX-M-14_ failed to transfer in both conjugation transfer assays. As indicated by previous studies, the *bla*_CTX-M-14_ can be located on the chromosome [88], which leads to failure of its horizontal transfer to *E. coli* J53. Research conducted on *bla*_CTX-M_ genes located on the chromosome has indicated that these genes are non-transferable to *E. coli* J53 [89]. In our study, *bla*_CTX-M-14_ failed to transfer, possibly due to its chromosomal location. In the present study, six out of eight ESBL-*E. coli* isolates capable of conjugative transfer were found to co-transfer *IS26* with *bla*_CTX-M_. *bla*_CTX-M_ genes are often co-localized with *IS26* on plasmids, a configuration that plays a key role in their mobilization and dissemination [90]. Consequently, it can be deduced that transfer of *bla*_CTX-M_ in isolates ESBLs01, ESBLs04, ESBLs08, ESBLs14, ESBLs15 and ESBLs17 may be mediated by *IS26* [91]. PBRT showed that the conjugative plasmids of ESBL-*E. coli* included IncFII, IncW, IncFrepB, IncY and IncHI1. These incompatibility-group types have been previously detected in ESBL-*E. coli* plasmids worldwide [92,93,94]. To the best of our knowledge, among ESBL-*E. coli* isolated from wildlife, the IncW plasmid has only been reported in wild birds prior to the present study [82]. The results of the present study demonstrate the presence of IncW plasmids in ESBL-*E. coli* from captive black bears, thus indicating a potential expansion of its host range within wildlife species. ESBL-*E. coli* with HGT capability poses a serious threat to public health by enabling dissemination across the interfaces between humans, animals and the environment, which is a core concern of the One Health framework [95,96]. The Asiatic black bear (*U. t. mupinensis*), the most widely distributed subspecies in China [15], may be a potential dissemination host due to its extensive contact with all three interfaces.

The limitations of this article are as follows: (1) small sample size and limited geographic scope of sampling; future studies should expand both the sample size and the geographic coverage of sampling; (2) environmental samples and samples from animal keepers were not collected; analyzing multiple interfaces enables a more comprehensive understanding of the transmission mechanisms. Nevertheless, the detection of ESBL-*E. coli* with HGT capabilities in black bears provides valuable insights into its dissemination potential, despite these limitations.

## 5. Conclusions

This study reports the first identification of ESBL-*E. coli* in captive black bears, with the isolates belonging to multiple different STs. The ESBL-*E. coli* isolates exhibited strong AMR and a diversity of resistance patterns. Furthermore, *bla*_CTX-M_ genes play a dominant role in mediating ESBL resistance. Conjugative transfer assays have demonstrated high transfer efficiency, and have shown that multiple ARGs, MGEs, and plasmids were capable of horizontal transmission. Based on the One Health framework and the needs of conservation, it is recommended that long-term surveillance be implemented to study the dissemination of ESBL-*E. coli* from captive black bears.

## Figures and Tables

**Table 1:** MLST typing results of 18 ESBL-*E. coli*.

Strains	ST	Clonal Complex
ESBLs01	nST1	/
ESBLs02	ST208	CC10
ESBLs03	ST10	CC10
ESBLs04	ST10	CC10
ESBLs05	ST695	None
ESBLs06	ST10	CC10
ESBLs07	ST10	CC10
ESBLs08	ST10	CC10
ESBLs09	ST4160	None
ESBLs10	ST10	CC10
ESBLs11	ST10	CC10
ESBLs12	nST2	/
ESBLs13	ST2690	None
ESBLs14	ST540	None
ESBLs15	*	*
ESBLs16	ST3856	None
ESBLs17	ST2792	None
ESBLs18	ST2690	None
ESBLs19	nST3	/

“*” represents that one housekeeping gene failed sequencing and was excluded from the analysis. “/” represents that ST could not be assigned based on database analysis; therefore, clonal complex was not determined.

**Table 2:** Comprehensive data of 19 ESBL-*E. coli* isolates from captive black bears.

Strains	Phenotypes	Variants of β-Lactam Resistance Genes	MGEs
ESBLs01	KZ, CRO, ATM	*bla_CTX-M-55_*	*IS26*, *trbC*, *ISECP1*
ESBLs02	KZ		*IS26*
ESBLs03	AMP, KZ, CXM, CRO, CTX, ATM, TZP	*bla_CTX-M-15_*	*IS26*, *ISECP1*
ESBLs04	AMP, KZ, CXM, CRO, CTX, ATM	*bla_CTX-M-15_*	*IS26*
ESBLs05	KZ, TZP		
ESBLs06	KZ, CXM, CRO, CTX, ATM	*bla_CTX-M-15_*	*IS26*, *trbC*, *ISECP1*
ESBLs07	AMP, KZ, CXM, CRO, CTX, FEP, ATM	*bla_CTX-M-15_*	*IS26*, *ISECP1*
ESBLs08	AMP, KZ, CXM, CRO, CTX, FEP, ATM	*bla_CTX-M-15_*	*IS26*, *ISECP1*
ESBLs09	AMP, KZ, CRO, CTX, ATM	*bla_CTX-M-15_*	*IS26*, *ISECP1*
ESBLs10	AMP, KZ, CXM, CRO, CTX, ATM	*bla_CTX-M-15_*	*IS26*, *ISECP1*
ESBLs11	AMP, KZ, CXM, CRO, CTX	*bla_CTX-M-15_*	*IS26*
ESBLs12	AMP, KZ, CXM, CRO, CTX, ATM	*bla_CTX-M-15_*	*IS26*
ESBLs13	AMP, KZ, CXM, CRO, CTX	*bla_CTX-M-27_*	*IS26*
ESBLs14	AMP, KZ, CXM, CRO, CTX	*bla_CTX-M-15_*, *bla_CTX-M-14_*	*IS26*, *tnpA*, *ISECP1*
ESBLs15	AMP, KZ, CRO, CTX, TZP	*bla_CTX-M-3_*, *bla_SHV-1_*	*IS26*, *trbC*
ESBLs16	KZ, IMP		*IS26*, *tnpA*, *tnsA*
ESBLs17	AMP, KZ, CXM, FOX, CRO, CTX, FEP, ATM, TZP	*bla_CTX-M-3_*, *bla_SHV-__1_*	*IS26*, *trbC*, *tnpA*, *tnsA*
ESBLs18	KZ, CXM, CRO, CTX, FEP, ATM, TZP	*bla_CTX-M-3_*, *bla_CTX-M-14_*, *bla_DHA-14_*	*IS26*, *trbC*, *tnpA*
ESBLs19	AMP, KZ, CXM	*bla_TEM-1_*	*IS26*, *trbC*, *tnpA*

## Data Availability

The original contributions presented in this study are included in the article/Supplementary Material. Further inquiries can be directed to the corresponding author.

## Supplementary Materials

The following supporting information can be downloaded at: https://www.mdpi.com/article/10.3390/vetsci12111085/s1, Table S1: PCR primer and conditions used in this study.
